# Bidirectional Risk Association Between Hidradenitis Suppurativa and Inflammatory Bowel Disease

**DOI:** 10.2340/actadv.v105.43657

**Published:** 2025-07-03

**Authors:** Louise LÖNNDAHL, Maria NAJEEB, Hassan KILLASLI, Lennart EMTESTAM, Ralf J. LUDWIG, Philip CURMAN

**Affiliations:** 1 Dermato-Venereology Clinic, Karolinska University Hospital, Stockholm; 2 Dermatology and Venereology Division, Department of Medicine (Solna), Karolinska Institutet, Stockholm; 3 Section of Infectious Disease and Dermatology, Department of Medicine, Huddinge, Karolinska Institutet, Stockholm, Sweden; 4 Institute and Comprehensive Centre for Inflammation Medicine, University-Hospital Schleswig-Holstein (UKSH), Campus Lübeck, Lübeck; 5 Department of Dermatology, UKSH, Campus Lübeck, Lübeck, Germany; 6 Lübeck Institute of Experimental Dermatology, University of Lübeck, Lübeck, Germany; 7 Department of Medical Epidemiology and Biostatistics, Karolinska Institutet, Stockholm, Sweden

**Keywords:** hidradenitis suppurativa, inflammatory bowel disease, Crohn’s disease, ulcerative colitis, matched cohort study, TriNetX

## Abstract

Hidradenitis suppurativa is an inflammatory skin disease of the pilosebaceous unit encompassing painful, recurrent, and malodorous abscesses, inflammatory nodules, dermal sinus tracts, and cicatrices. Hidradenitis suppurativa has been linked to several comorbidities, including inflammatory bowel disease, though evidence is limited by small sample sizes and possible bidirectional associations remain uncertain. A large-scale, propensity-score matched retrospective cohort study was performed using the TriNetX platform to investigate bidirectional risks between hidradenitis suppurativa and inflammatory bowel disease. Data included over 200,000 hidradenitis suppurativa patients and 3.3 million inflammatory bowel disease patients. Hidradenitis suppurativa was associated with a 36% increased risk of developing any inflammatory bowel disease diagnosis within 5 years of diagnosis (hazard ratio [HR] 1.356, confidence interval [CI] 1.313–1.401), with the highest risk of Crohn’s disease (HR 2.501, CI 2.209–2.832), followed by ulcerative colitis (UC) (HR 1.722, CI 1.536–1.930), and other/unspecified inflammatory bowel disease (HR 1.349, CI 1.306–1.394). Conversely, individuals with any inflammatory bowel disease diagnosis had a 31% increased risk of developing hidradenitis suppurativa (HR 1.313, CI 1.274–1.353), with Crohn’s disease showing the strongest association (HR 2.441, CI 2.242–2.658), followed by UC (HR 1.493, CI 1.358–1.642). These findings highlight the bidirectional risk relationship between hidradenitis suppurativa and inflammatory bowel disease, demonstrating that the likelihood of developing either condition is significantly elevated in patients already diagnosed with the other.

## SIGNIFICANCE

Hidradenitis suppurativa has been linked to inflammatory bowel disease, but previous studies have been limited by small sample sizes, and the reverse association remained uncertain. Our findings show a strong bidirectional risk relationship between HS and inflammatory bowel disease, with the largest risks between hidradenitis suppurativa and Crohn’s disease. The clinical implication of our study highlights the need for a multidisciplinary approach involving both dermatologists and gastroenterologists in order to facilitate early diagnosis and intervention in patients with hidradenitis suppurativa or inflammatory bowel disease to reduce unnecessary patient suffering.

Hidradenitis suppurativa (HS) is an inflammatory skin disease of the pilosebaceous unit encompassing painful, recurrent, and malodorous abscesses, inflammatory nodules, dermal sinus tracts, and cicatrices in the affected anatomical regions. More specifically, this non-communicable chronic inflammatory disease predominantly occurs in areas with high concentrations of sebaceous glands, including the axilla, intergluteal cleft, groin, and inframammary region. The estimated global burden of HS reaches a prevalence of 4.1% (1), with a reported prevalence between 0.7% and 1.2% in Europe and the United States (2). The exact pathogenesis remains to be elucidated,, with suggested mechanisms involving genetic predisposition, environmental, inflammatory, and bacterial factors. HS has been linked to a plethora of comorbidities, including inflammatory bowel disease (IBD). Insights into reciprocal risks are, however, uncertain, and are important for patient management.

The chronic inflammation of the gastrointestinal tract known as IBD can be classified into 2 main forms: ulcerative colitis (UC) and Crohn’s disease (CD). The clinical presentation of UC includes bloody stools, diarrhoea, abdominal pain, pyrexia, and anaemia. In addition to these symptoms, CD can present with extraintestinal manifestations including inflammation of the skin.

In recent years, there has been a growing concern to understand the potential relationship between HS and IBD. Previous studies have indicated a significant association between HS and both CD and UC. A systematic review and meta-analysis reviewed 5 case-control studies, 2 cross-sectional studies, and 1 cohort study, encompassing in total 93,601 patients, which illustrated significant associations of HS with CD and UC (3). This is further reinforced by another meta-analysis that identified a noteworthy prevalence of IBD in HS patients, more specifically seen with CD and HS (4).While some individual studies have reported inconsistent findings (5, 6), meta-analyses such as that by Phan et al. (4) have demonstrated significant associations between HS and both CD and UC, supporting a consistent overall link. However, these individual studies are hampered by their relatively small sample sizes and the lack of evaluating time-dependent risks, because patients with HS and IBD experience significant diagnostic delay, suggests that it may remain unclear which diagnosis occurred first (7, 8).

To address these clinically important knowledge gaps, this study investigates the bidirectional risks between HS and IBD using electronic health records (EHRs) from a large-scale federated US database comprising over 200,000 HS and over 3.3 million IBD patients. The risk of developing IBD after a documented diagnosis of HS and, conversely, the risk of developing HS after IBD is examined. This aims to provide clinically and mechanistically relevant insights regarding these reciprocal comorbidity risks.

## METHODS

### Study design and database

We performed a large-scale retrospective propensity-score matched cohort study utilizing the global federated TriNetX platform using established protocol (9–12). Data from the US Collaborative Network with, at the time of analysis, access to over 120 million EHRs from 69 health care organizations (HCOs) in the USA was used. Data were retrieved in December 2024. Five patient cohorts, described in detail below and in **Table I**, were defined: (*i*) HS, (*ii*) any IBD, (*iii*) CD or UC, (*iv*) CD, and (*v*) UC. Each patient cohort was compared with a respective control group. The index event, defining the entry of each participant in the study, was defined as a diagnosis of either disease listed above (patients) or a diagnosis of ICD-10CM:Z00 “Encounter for general examination without complaint, suspected or reported diagnosis” (controls). Individuals aged 15 or older at the index event were considered.

**Table I:** Baseline characteristics of all study cohorts before propensity-score matching

Characteristic	Before propensity-score matching			
HS vs controls	HS	Controls	*p*-value	Std diff.
*n*	203,966	13,883,932	–	–
Age at index (years, SD)	36.4 ± 14.5	44.4 ± 19.2	< 0.001	0.468
White (%)	46.6%	60.8%	< 0.001	0.289
Female (%)	73.0%	52.0%	< 0.001	0.445
BMI (mean, SD)	34.7 ± 9.1	28.6 ± 7.3	< 0.001	0.731
Personal history of nicotine dependence (%)	5.7%	4.2%	< 0.001	0.069
Nicotine dependence (%)	16.5%	5.4%	< 0.001	0.361
Family history of diseases of the digestive system (%)	0.4%	0.3%	< 0.001	0.023
Any IBD vs controls	Any IBD	Controls	*p*-value	Std diff.
*n*	3,341,433	12,708,427	–	–
Age at index (years, SD)	47.2 ± 19.1	44.1 ± 19.1	< 0.001	0.162
White (%)	65.5%	60.1%	< 0.001	0.110
Female (%)	56.7%	51.8%	< 0.001	0.100
BMI (mean, SD)	29.0 ± 7.5	28.6 ± 7.3	< 0.001	0.044
Personal history of nicotine dependence (%)	6.9%	3.8%	< 0.001	0.137
Nicotine dependence (%)	10.4%	5.0%	< 0.001	0.205
Family history of diseases of the digestive system (%)	0.6%	0.2%	< 0.001	0.049
CD or UC vs controls	CD or UC	Controls	*p*-value	Std diff.
*n*	631,573	13,774,037	–	–
Age at index (years, SD)	47.1 ± 18.6	44.3 ± 19.2	< 0.001	0.150
White (%)	70.0%	60.6%	< 0.001	0.198
Female (%)	52.1%	52.1%	0.164	0.002
BMI (mean, SD)	28.0 ± 7.0	28.7 ± 7.3	< 0.001	0.095
Personal history of nicotine dependence (%)	5.3%	4.1%	< 0.001	0.056
Nicotine dependence (%)	6.3%	5.4%	< 0.001	0.041
Family history of diseases of the digestive system (%)	0.9%	0.3%	< 0.001	0.080
CD vs controls	CD	Controls	*p*-value	Std diff.
*n*	336,141	13,873,232	–	–
Age at index (years, SD)	43.9 ± 18.3	44.3 ± 19.2	< 0.001	0.024
White (%)	70.7%	60.7%	< 0.001	0.213
Female (%)	53.1%	52.1%	< 0.001	0.019
BMI (mean, SD)	27.5 ± 7.1	28.7 ± 7.3	< 0.001	0.160
Personal history of nicotine dependence (%)	4.1%	4.2%	0.005	0.005
Nicotine dependence (%)	5.9%	5.4%	< 0.001	0.022
Family history of diseases of the digestive system (%)	1.0%	0.3%	< 0.001	0.092
UC vs controls	UC	Controls	*p*-value	Std diff.
*n*	352,670	13,845,637	–	–
Age at index (years, SD)	49.7 ± 18.4	44.3 ± 19.2	< 0.001	0.291
White (%)	69.7%	60.7%	< 0.001	0.190
Female (%)	50.7%	52.2%	< 0.001	0.029
BMI (mean, SD)	28.1 ± 6.9	28.7 ± 7.3	< 0.001	0.076
Personal history of nicotine dependence (%)	7.2%	4.2%	< 0.001	0.133
Nicotine dependence (%)	7.4%	5.4%	< 0.001	0.081
Family history of diseases of the digestive system (%)	1.0%	0.3%	< 0.001	0.089

After matching, no major differences were found in any of the matching variables. As body mass index (BMI) is a continuous variable with known variability, pre- and post-matching values were similar between groups.

CD: Crohn’s disease; HS: hidradenitis suppurativa; IBD: inflammatory bowel disease; SD: standard deviation; Std diff: standardized difference; UC: ulcerative colitis.

### Study population, definition of eligible study participants, and outcomes

As indicated above, 5 patient cohorts were defined. We retrieved (*i*) 203,966 patients with HS (ICD-10CM:L73.2), (*ii*) 3,341,433 patients with any IBD diagnosis (ICD-10CM:K50-K52), (*iii*) 631, 573 patients with CD or UC (ICD-10CM:K50-K51), (*iv*) 336,141 patients with CD (ICD-10CM:K50), and (v) 35,267 patients with UC (ICD-10CM:K51). Controls (*n* = 13,883,932) were defined by the exclusion of the respective diagnosis of the patient group with which it was compared. For example, HS patients were compared with controls without any diagnosis of HS (ICD-10CM:L73.2) and any IBD were compared with controls without any diagnosis of IBD (ICD-10CM:K50-K52), etc. (Table I and Table SI). All 69 HCOs responded with available EHRs.

The outcomes for (*i*) the HS cohort were defined as any IBD (ICD-10CM:K50-K52), CD (ICD-10CM:K50), UC (ICD-10CM:K51), and other/unspecified noninfective gastroenteritis and colitis (ICD-10CM:K52), whereas the outcome for (*ii*–*v*) the IBD cohorts was defined as a diagnosis of HS (ICD-10CM:L73.2).

### Covariates

Propensity-score matching (PSM) was performed to balance the cohorts to optimize comparability and to address bias imposed by potential confounding covariates. A covariate matrix for PSM was established, including demographic information and possible risk factors for IBD and HS, respectively, covering the following covariates: age at index (continuous variable), white race (binary), female sex (binary), body mass index (BMI, kg/m^2^), personal history of nicotine dependence (ICD-10CM:Z87.891, binary), nicotine dependence (ICD-10CM:F17, binary), and family history of diseases of the digestive system (ICD-10CM:Z83.7, binary).

In sensitivity analysis S3 (see below), an extended PSM was performed, where the following covariates were added: diseases of the circulatory system (ICD-10CM:I00-I99, binary), endocrine, nutritional and metabolic diseases (ICD-10CM:E00-E89, binary), diseases of the musculoskeletal system and connective tissue (ICD-10CM:M00-M99, binary), antimicrobial medications (Veteran Affairs National Formulary [VA], using RxNorm codes for individual drugs:AM000), musculoskeletal medications (VA:MS000), and immunological agents (VA:IM000; Table SII). It should be noted that for the extended PSM in S3 the cohort sizes had to be reduced for the number of matching variables to be successful. This was done by setting the cut-off for data retrieval to June 2019. The matrix row order was randomized after data retrieval. A propensity score for each patient was generated by logistic regression analysis (with exposure as the dependent variable) using the Python package scikit-learn (https://scikit-learn.org/stable/). Matching was performed 1:1 using the greedy nearest neighbour approach with a cut-off distance of 0.1 pooled standard deviations of the logit of the propensity score. Baseline characteristics were re-evaluated and reported after matching; differences were compared by *t*-test for continuous and z-test for binary or categorical variables.

### Statistical analysis

The primary analysis investigated outcomes 1 day to 5 years after the index event for all comparisons. Additionally, for the risk relationship between HS and any IBD, both a matched and crude/unmatched analysis was performed. Our results were contested in 3 sensitivity analyses: (S1) outcomes 1–5 years after the index event to mitigate detection bias, (S2) outcomes 1 day to any time after index to assess longer-term risks, and (S3) outcomes 1 day to 5 years after index with extended PSM criteria to account for additional possible confounders (Table SII). Sensitivity analyses S1–S2 were performed for all comparisons, while sensitivity analysis S3 was performed only for the HS cohort with IBD outcomes. Due to the significantly larger size of the IBD cohort compared with HS, applying the extended PSM to IBD-to-HS analyses would have required excessive data reductions, potentially limiting statistical power and generalizability. Any outcomes prior to the index event were excluded in all analyses after PSM. Survival analyses were performed using the Kaplan–Meier method (KM). The proportionality assumption was tested by the coxph function in R’s Survival package (R Foundation for Statistical Computing, Vienna, Austria), using Schoenfeld residuals and χ^2^ tests. KM curves were compared using the log-rank test. A univariate Cox proportional hazards regression was used to express hazard ratios (HR)s with 95% confidence intervals (CI)s.

## RESULTS

### Study population characteristics

**Fig 1:**
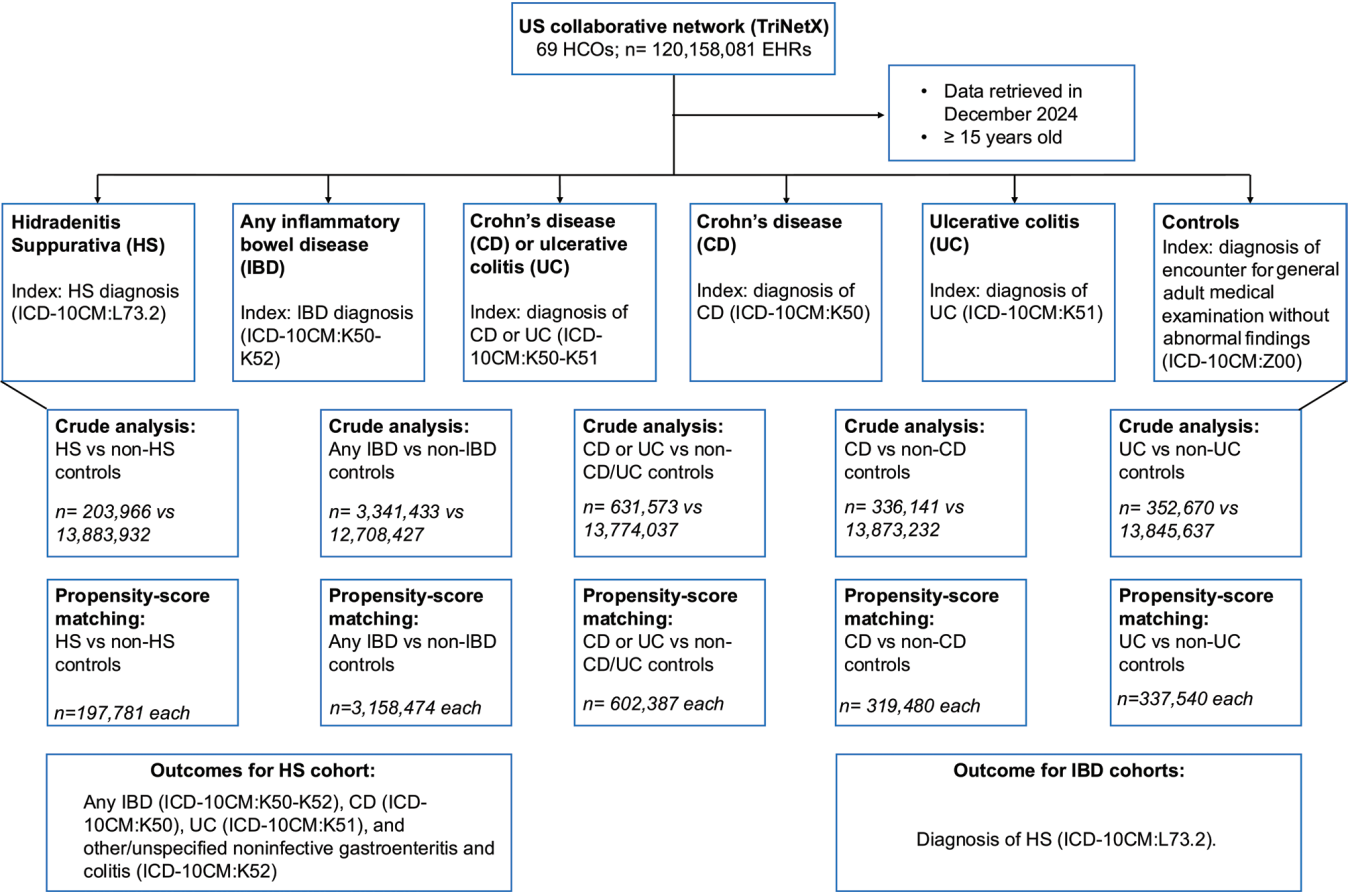
Study flowchart.

Data from a total of 120,158,081 patients were screened. For the primary analyses, the number of EHRs in the study cohorts after 1:1 PSM were (*i*) 197,781 each for “HS” and controls, (*ii*) 3,158,474 each for “any IBD” and controls, (*iii*) 602,387 each for “CD or UC” and controls, (*iv*) 319,480 each for “CD” and controls, and (*v*) 337,540 for “UC” and controls (**Fig. 1**). After PSM, no major differences were found in any of the matching variables. As BMI is a continuous variable with known variability, pre- and post-matching values were similar between groups (Table I and Table SII). The follow-up times (mean, SD) after PSM in the respective comparison groups for the primary analyses were (*i*) 1,020 ± 712 and 1,039 ± 721 days, (*ii*) 1,063 ± 740 and 1,042 ± 716 days, (*iii*) 1,031 ± 733 and 1,078 ± 719 days, (*iv*) 1,057 ± 737 and 1,076 ± 723 days, and (*v*) 1,031 ± 722 and 1,082 ± 714 days for cases and controls, respectively.

### Increased risk of developing IBD after a diagnosis of HS compared with controls

The crude, unmatched analysis of the association between HS and the risk of developing any IBD diagnosis reveals a 41% increased risk within 5 years of HS diagnosis (HR 1.405, CI 1.374–1.436, *p* < 0.001). Most prominently, there is a 2.6 times risk of developing CD (HR 2.624, CI 2.451–2.809, *p* < 0.001), while the risk of UC is 47% (HR 1.467, CI 1.367–1.574, *p* < 0.001) and the risk of other/unspecified IBD is increased by 41% (HR 1.413, CI 1.382, 1.444, *p* < 0.001).

**Fig 2:**
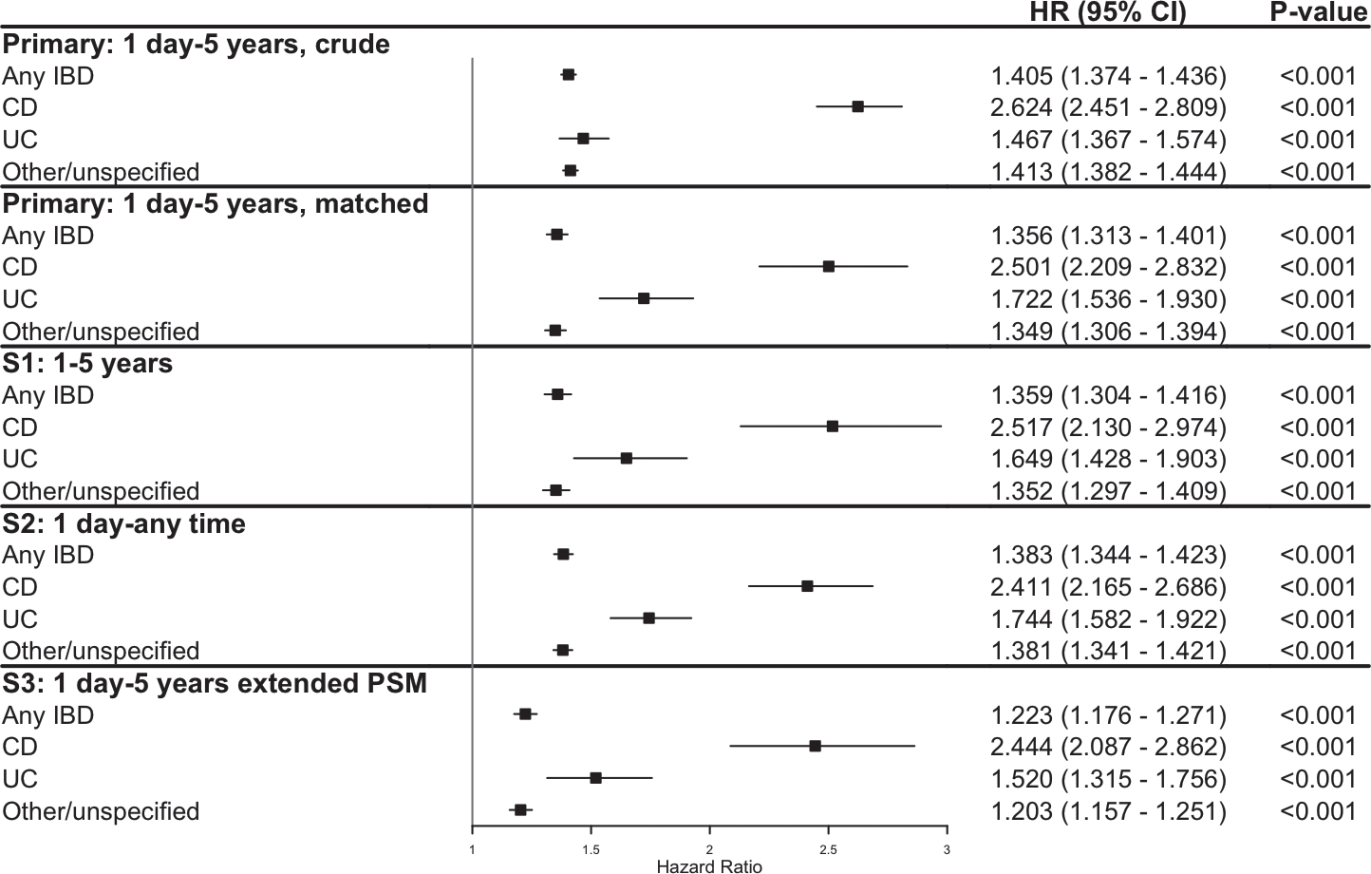
**Risk of inflammatory bowel disease (IBD) following a diagnosis of hidradenitis suppurativa (HS).** Data presented as hazard ratio (HR) with 95% confidence intervals (CI) for primary and sensitivity analyses. CD: Crohn’s disease; PSM: propensity-score matching; UC: ulcerative colitis.

The elevated risk of developing IBD in HS patients remains in the matched cohorts. The risk of developing any IBD is elevated at 36% (HR 1.356, CI 1.313–1.401, *p* < 0.001). For CD, the risk remains increased by 2.5 times (HR 2.501, CI 2.209–2.832, *p* < 0.001), and the risk of UC is further increased to 72% (HR 1.722, CI 1.536–1.930, *p* < 0.001). A 35% increased risk for other or unspecified IBDs is also seen (HR 1.349, CI 1.306–1.394, *p* < 0.001). The results were consistent when removing any outcomes in the first year after diagnosis in sensitivity analysis S1, when extending follow-up to any time after diagnosis in sensitivity analysis S2, and when performing an extended PSM for outcomes within 5 years in sensitivity analysis S3 (**Fig. 2** and Table SIII).

### Increased risk of developing HS after a diagnosis of IBD compared with controls

**Fig 3:**
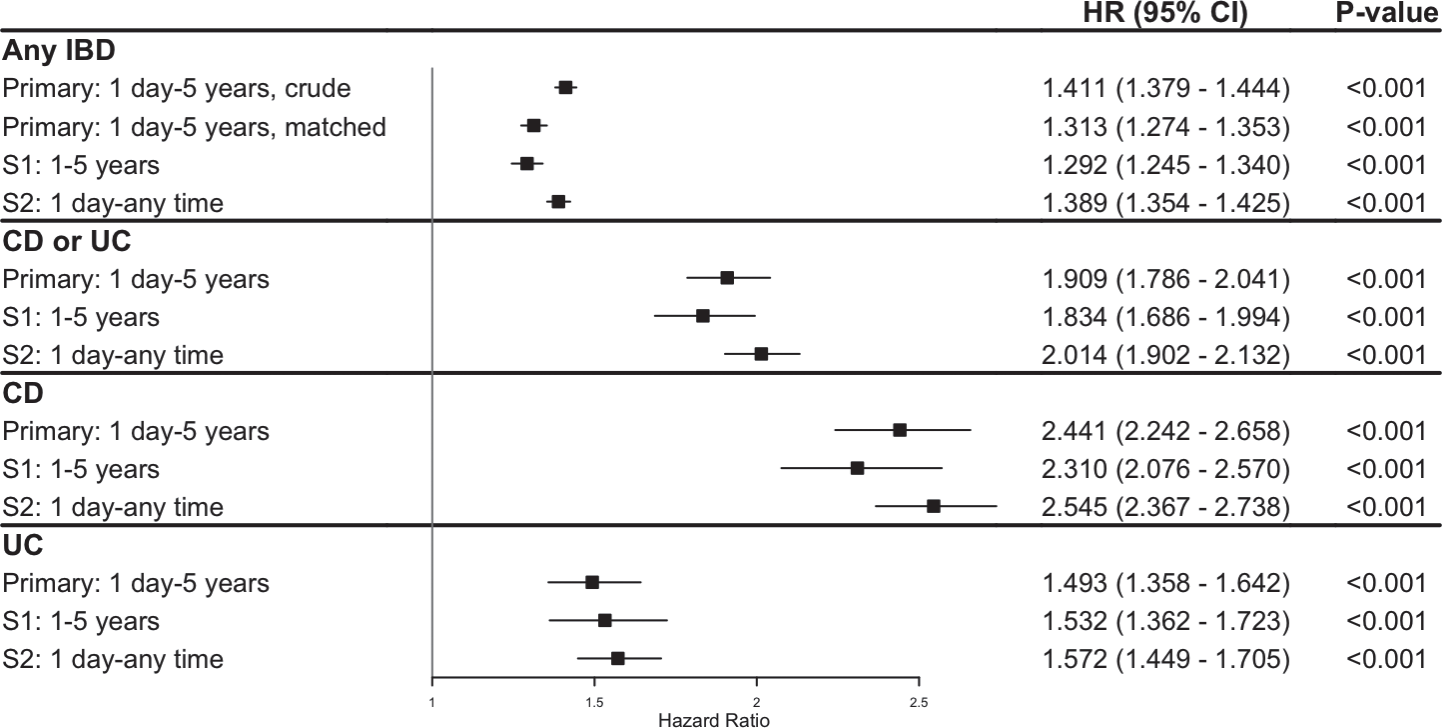
**Risk of hidradenitis suppurativa (HS) following a diagnosis of inflammatory bowel disease (IBD).** Data presented as hazard ratio (HR) with 95% confidence intervals (CI) for primary and sensitivity analyses. CD: Crohn’s disease; PSM: propensity-score matching; UC: ulcerative colitis.

Individuals with any IBD diagnosis were found to have a 41% increased risk of developing HS within 5 years in the unmatched analysis (HR 1.411, CI 1.379–1.444, *p* < 0.001). Similarly, the risk remained elevated by 31% when adjusted for confounding factors using PSM (HR 1.313, CI 1.274–1.353, *p* < 0.001). Individuals with a diagnosis of CD or UC had a 1.9-fold increased risk (HR 1.909, CI 1.786–2.041, *p* < 0.001), while the risk for individuals with CD was elevated 2.4 times (HR 2.441, CI 2.242–2.658, *p* < 0.001). Individuals with UC exhibited a 49% increased risk (HR 1.493, 1.358–1.642, *p* < 0.001). Both sensitivity analyses S1 and S2 showed comparable results for all comparison groups (**Fig. 3** and Table SIV).

## DISCUSSION

This study underscores the bidirectional risk relationship between HS and IBD, revealing a significantly elevated risk for developing one condition in patients already diagnosed with the other. To ensure the robustness of our results, we conducted 3 sensitivity analyses. The relationship was particularly pronounced between HS and CD, although a notable and significant association was also evident with UC.

### Bidirectional risk relationship between HS and IBD

Given the chronic inflammatory nature of both HS and IBD, we sought to explore the reciprocal risk of developing one condition in patients already diagnosed with the other, rather than limiting our analysis to relative risks or odds ratios. This approach allowed us to account for the timing of diagnoses and consider the potential overlap of low-grade symptoms that might not yet lead to or warrant a formal diagnosis but still indicate underlying disease activity. Both HS and IBD are prone to diagnostic delays, and our time-to-event analyses help capture the true risk regardless of when a formal diagnosis occurs (7, 13).

While these diagnostic delays make it difficult to determine which condition typically precedes the other, our consistent finding of elevated risk in both directions underscores that the primary focus should not be on which disease arises first, but on the heightened overall risk for affected patients. This has critical clinical implications, emphasizing the importance of a multidisciplinary approach that integrates both dermatology and gastroenterology to ensure timely diagnosis and intervention.

### Shared genetic and immunological mechanisms

The similarities between the diseases are of considerable importance. Both conditions affect barrier tissues, share components of activated immunological pathways, and may have a shared genetic predisposition.

Several studies have previously indicated genetic associations that increase the risk for HS and IBD (14, 15). However, there is limited literature investigating the relationship between specific genes and the coexistence of these 2 conditions. One study investigating patients with both IBD and HS found a possible connection to the genes SULT1B1 (OMIM 608436) and SULT1E1 (OMIM 600043) (16). Interestingly, the SULT1E1 gene encodes an enzyme involved in oestrogen homeostasis, which might suggest a link to disease development given the female predominance of HS and the influence of hormonal factors on disease onset and flares. Moreover, studies have reported that a large proportion of patients (34–36%) with HS also have affected family members (17). Similarly, for IBD, family history of the disease also seems important, with a 4–8-fold risk increase for first-degree relatives (18).

While genetic predisposition may underlie both diseases, the directionality of risk remains debated. Bao et al. recently applied bidirectional Mendelian randomization and found genetic evidence supporting a causal effect of IBD on HS, but not the reverse (19). This may reflect methodological differences, as Mendelian randomization is limited by genetic pleiotropy and may not fully capture environmental or clinical factors addressed in large-scale, real-world cohort studies. In contrast, the bidirectional nature of our findings suggests that both directions of risk are clinically relevant.

Both conditions share elevated levels of proinflammatory cytokines, namely tumour necrosis factor (TNF)-alfa, interleukin (IL)-1β, IL-12, IL-17, IL-23, and complement (20, 21). In addition, anti-TNF-α therapy remains the most commonly used treatment for both conditions, indicating activation of shared inflammatory pathways. Notably, the IL-17 pathway has emerged as a key target in HS, with IL-17 inhibitors showing increasing efficacy, further underscoring the immunological overlap with IBD where IL-17 is also implicated (22). These shared cytokine profiles also point to overlapping therapeutic targets, including TNF-α, IL-12/23, and IL-17, that may be leveraged for dual treatment strategies in patients with comorbid disease. Additionally, the microbiome plays a crucial role in both inflammatory conditions, with potential links to inflammatory response and interactions between the 2 (23). Recent studies have demonstrated that HS lesions are characterized by a distinct dysbiosis, with altered bacterial communities paralleling some of the changes observed in IBD, suggesting a shared microbial influence on disease pathogenesis (24). Given that both diseases affect barrier tissues and modify the microbiota during disease progression, this connection is of great interest (25). It is noteworthy that the role of other associated risk factors such as smoking and obesity, as discussed by Jiang et al., as well as the possible effects of pharmacological treatment on inflammatory response and microbiota remains to be investigated (21). The connection between the diseases is indeed of great immunological interest. For instance, studies have shown loss of function in Notch signalling, which plays a role in regulating cell proliferation in the hair follicles, potentially explaining its link to HS (26, 27).

### Strengths and limitations

The primary strength of this study is the utilization of an extensive dataset, encompassing over 120 million EHRs. The large cohort sizes facilitate robust PSM, significantly reducing bias from potential confounders and enhancing the reliability of the results. Additionally, the rigorous sensitivity analyses performed challenge our findings and ensure consistency across various scenarios, adding to the robustness of the study. The comprehensive data source provided by the TriNetX platform allows access to a diverse and representative sample, enhancing the generalizability of our findings.

Despite these strengths, several limitations must be acknowledged. As with any study relying on EHR data, there is an inherent risk of miscoding, undercoding, or misclassification of diagnoses, which could affect the results. The observational nature of the study precludes the establishment of causal relationships between HS and IBD. Another important consideration is the potential for diagnostic delays, which are common in both HS and IBD (7, 13). These delays could introduce timing bias if symptoms of one disorder were present before a formal diagnosis was made. Nevertheless, the bidirectional risk observed mitigates this concern, suggesting that the overall findings remain robust despite this potential bias. Lastly, despite the use of PSM, some residual confounding may still be present.

### Conclusion

Tthe significant increase in the risk of developing IBD after an HS diagnosis, as well as the heightened risk of developing HS after an IBD diagnosis, emphasizes the likely shared pathophysiological and immunological mechanisms driving disease development. In light of these findings, we emphasize the importance of ongoing and future research into inflammatory diseases affecting barrier tissues. The consistent finding of elevated risk in both directions suggests that the focus should not be on which disease develops first, but rather on the overall bidirectional risk imposed on patients with either condition. This is of great clinical importance and highlights the need for a multidisciplinary approach involving both dermatologists and gastroenterologists to ensure timely diagnosis and intervention, thereby minimizing unnecessary patient suffering.

## Supplementary Material


