# Supplementary material for: Bidirectional Risk Association Between Hidradenitis Suppurativa and Inflammatory Bowel Disease
Source: Acta Derm Venereol. 2025 Jul 3;105:43657. doi: 10.2340/actadv.v105.43657 (PMC12239007; doi:10.2340/actadv.v105.43657)
Supplement: Supplementary file 1 [file ActaDV-105-43657-s1.pdf]

Supplementary material has been published as submitted. It has not been copyedited, or typeset by Acta Dermato-Venereologica

Table SI. Cohort definitions

**Hidradenitis suppurativa**	**Controls**
Inclusion criteria<br><br>Hidradenitis suppurativa (UMLS:ICD10CM:L73.2) (at least 15 years old at event).	***Inclusion criteria***<br><br>Encounter for general examination without complaint, suspected or reported diagnosis (UMLS:ICD10CM:Z00) (at least 15 years old at event).<br><br>***Exclusion criteria***<br><br>Hidradenitis suppurativa (UMLS:ICD10CM:L73.2).
**Any inflammatory bowel disease**	**Controls**
Inclusion criteria<br><br>Noninfective enteritis and colitis (UMLS:ICD10CM:K50-K52) (at least 15 years old at event).	***Inclusion criteria***<br><br>Encounter for general examination without complaint, suspected or reported diagnosis (UMLS:ICD10CM:Z00) (at least 15 years old at event).<br><br>***Exclusion criteria***<br><br>Noninfective enteritis and colitis (UMLS:ICD10CM:K50-K52)
**Crohn's disease or ulcerative colitis**	**Controls**
Inclusion criteria<br><br>Crohn's disease [regional enteritis] (UMLS:ICD10CM:K50) (at least 15 years old at event); or<br>Ulcerative colitis (UMLS:ICD10CM:K51) (at least 15 years old at event).	***Inclusion criteria***<br><br>Encounter for general examination without complaint, suspected or reported diagnosis (UMLS:ICD10CM:Z00) (at least 15 years old at event).<br><br>***Exclusion criteria***<br><br>Crohn's disease [regional enteritis] (UMLS:ICD10CM:K50); or Ulcerative colitis (UMLS:ICD10CM:K51).
**Crohn's disease**	**Controls**
Inclusion criteria<br><br>Crohn's disease [regional enteritis] (UMLS:ICD10CM:K50) (at least 15 years old at event)	***Inclusion criteria***<br><br>Encounter for general examination without complaint, suspected or reported diagnosis (UMLS:ICD10CM:Z00) (at least 15 years old at event).<br><br>***Exclusion criteria***<br><br>Crohn's disease [regional enteritis] (UMLS:ICD10CM:K50).

Ulcerative colitis	Controls
Inclusion criteria<br><br>Ulcerative colitis (UMLS:ICD10CM:K51) (at least 15 years old at event).	***Inclusion criteria***<br><br>Encounter for general examination without complaint, suspected or reported diagnosis (UMLS:ICD10CM:Z00) (at least 15 years old at event).<br><br>***Exclusion criteria***<br><br>Ulcerative colitis (UMLS:ICD10CM:K51).

Table SII. Baseline characteristics for sensitivity analysis S3 with extended propensity-score matching.

BMI, body mass index; HS, hidradenitis suppurativa; ICD-10CM, international classification of diseases 10th edition Clinical Modification; SD, standard deviation; Std. diff., standardized difference.

Characteristic		Before matching				After matching			
				P-value	Std. diff.			P-value	Std. diff.
		HS	Controls			HS	Controls		
n		99,787	7,537,931	-	-	93,601	93,601	-	-
Age at index (years, SD)		36.4 ± 14.4	43.8 ± 18.3	<0.001	0.446	36.4 ± 14.4	36.5 ± 14.5	0.448	0.004
White (%)		46.3%	61.3%	<0.001	0.304	46.3%	46.3%	0.889	0.001
Female (%)		72.4%	53.6%	<0.001	0.397	72.4%	72.5%	0.897	0.001
BMI (mean, SD)		34.0 ± 9.0	28.5 ± 7.1	<0.001	0.689	34.0 ± 9.0	29.7 ± 8.2	<0.001	0.511
**Diagnosis**	**ICD-10CM code**								
Personal history of nicotine dependence (%)	Z87.891	3.7%	2.6%	<0.001	0.061	3.7%	3.6%	0.192	0.006
Nicotine dependence (%)	F17	15.0%	4.3%	<0.001	0.369	15.0%	15.0%	0.836	0.001
Family history of diseases of the digestive system (%)	Z83.7	0.2%	0.2%	0.157	0.005	0.2%	0.2%	<0.001	0.017
Diseases of the circulatory system (%)	I00-I99 (entire chapter)	26.8%	22.9%	<0.001	0.091	26.8%	26.8%	0.917	<0.001
Endocrine, nutritional and metabolic diseases (%)	E00-E89 (entire chapter)	39.8%	28.1%	<0.001	0.248	39.8%	39.8%	0.817	0.001
Diseases of the musculoskeletal system and connective tissue (%)	M00-M99 (entire chapter)	41.0%	29.9%	<0.001	0.233	41.0%	41.0%	0.929	<0.001
**Medication**	**VA drug classification code**								
Antimicrobials (%)	AM000 (entire chapter)	50.3%	28.1%	<0.001	0.467	50.3%	50.3%	0.736	0.002
Musculoskeletal medications (%)	MS000 (entire chapter)	38.0%	20.1%	<0.001	0.402	38.0%	38.2%	0.452	0.003
Immunological agents (%)	IM000 (entire chapter)	21.8%	15.0%	<0.001	0.177	21.8%	21.8%	0.704	0.002

Table SIII. Detailed results for the risk of inflammatory bowel disease after a diagnosis of hidradenitis suppurativa.

CD, Crohn's disease; CI, confidence interval; HR, hazard ratio; IBD, inflammatory bowel disease; PSM, propensity-score matching; RR, risk ratio; UC, ulcerative colitis.

Outcome	Hidradenitis suppurativa		Control group		Hazard ratio (CI)	P-value HR
	N of eligible participants*	N with outcome	N of eligible participants*	N with outcome		
**Primary analysis, crude (1 day to 5 years after index event)**						
Any IBD	178,640	8,233	12,938,846	445,814	1.405 (1.374, 1.436)	<0.001
CD	194,524	857	13,482,356	23,337	2.624 (2.451, 2.809)	<0.001
UC	196,060	794	13,479,861	38,419	1.467 (1.367, 1.574)	<0.001
Other/unspecified	181,007	8,049	13,009,126	430,216	1.413 (1.382, 1.444)	<0.001
**Primary analysis, matched (1 day to 5 years after index event)**						
Any IBD	178,640	8,233	188,001	6,572	1.356 (1.313, 1.401)	<0.001
CD	194,524	857	196,837	351	2.501 (2.209, 2.832)	<0.001
UC	196,060	794	196,949	470	1.722 (1.536, 1.930)	<0.001
Other/unspecified	181,007	8,049	188,942	6,407	1.349 (1.306, 1.394)	<0.001
**Sensitivity analysis S1 (1-5 years after index event)**						
Any IBD	175,491	5,084	185,553	4,124	1.359 (1.304, 1.416)	<0.001
CD	194,138	471	196,681	195	2.517 (2.130, 2.974)	<0.001
UC	195,749	483	196,782	303	1.649 (1.428, 1.903)	<0.001
Other/unspecified	177,939	4,981	186,564	4,029	1.352 (1.297, 1.409)	<0.001
**Sensitivity analysis S2 (1 day to any time after index event)**						
Any IBD	178,640	10,515	188,001	8,453	1.383 (1.344, 1.423)	<0.001
CD	194,524	1,091	196,837	474	2.411 (2.165, 2.686)	<0.001
UC	196,060	1,079	196,949	647	1.744 (1.582, 1.922)	<0.001
Other/unspecified	181,007	10,282	188,942	8,213	1.381 (1.341, 1.421)	<0.001
**Sensitivity analysis S3 (1 day to 5 years after index event with extended PSM)**						
Any IBD	84,866	5,536	87,249	4,656	1.223 (1.176, 1.271)	<0.001
CD	92,169	530	93,028	217	2.444 (2.087, 2.862)	<0.001
UC	92,938	464	93,153	303	1.520 (1.315, 1.756)	<0.001
Other/unspecified	85,893	5,443	87,724	4,618	1.203 (1.157, 1.251)	<0.001

Table SIV. Detailed results for the risk of hidradenitis suppurativa after a diagnosis of inflammatory bowel disease.

CI, confidence interval; HR, hazard ratio; HS, hidradenitis suppurativa; IBD, inflammatory bowel disease; RR, risk ratio.

Analysis (outcome: HS)	IBD group		Control group		Hazard ratio (CI)	P-value HR
	N of eligible participants*	N with outcome	N of eligible participants*	N with outcome		
**Any IBD**						
Primary: 1 day-5 years, crude	3,145,628	9,992	12,390,582	27,249	1.411 (1.379, 1.444)	<0.001
Primary: 1 day-5 years, matched	3,145,628	9,992	3,149,748	7,518	1.313 (1.274, 1.353)	<0.001
S1: 1-5 years	3,142,248	6,612	3,147,188	4,958	1.292 (1.245, 1.340)	<0.001
S2: 1 day-any time	3,145,628	14,938	3,149,748	9,961	1.389 (1.354, 1.425)	<0.001
**Crohn's disease or ulcerative colitis**						
Primary: 1 day-5 years	599,902	2,435	601,026	1,333	1.909 (1.786, 2.041)	<0.001
S1: 1-5 years	598,968	1,501	600,554	861	1.834 (1.686, 1.994)	<0.001
S2: 1 day-any time	599,902	3,452	601,026	1,793	2.014 (1.902, 2.132)	<0.001
**Crohn's disease**						
Primary: 1 day-5 years	317,795	1,790	318,724	750	2.441 (2.242, 2.658)	<0.001
S1: 1-5 years	317,102	1,097	318,459	485	2.310 (2.076, 2.570)	<0.001
S2: 1 day-any time	317,795	2,552	318,724	1,014	2.545 (2.367, 2.738)	<0.001
**Ulcerative colitis**						
Primary: 1 day-5 years	336,217	1,031	336,729	724	1.493 (1.358, 1.642)	<0.001
S1: 1-5 years	335,866	680	336,475	470	1.532 (1.362, 1.723)	<0.001
S2: 1 day-any time	336,217	1,446	336,729	973	1.572 (1.449, 1.705)	<0.001
